# Factor H-related protein 1 (FHR-1) is associated with atherosclerotic cardiovascular disease

**DOI:** 10.1038/s41598-021-02011-w

**Published:** 2021-11-18

**Authors:** Sarah Irmscher, Svante L. H. Zipfel, Luke D. Halder, Lia Ivanov, Andres Gonzalez-Delgado, Christoph Waldeyer, Moritz Seiffert, Fabian J. Brunner, Monika von der Heide, Ina Löschmann, Sonia Wulf, Darina Czamara, Nikolina Papac-Milicevic, Olaf Strauß, Stefan Lorkowski, Hermann Reichenspurner, Michael V. Holers, Nirmal K. Banda, Tania Zeller, Elisabeth B. Binder, Christoph J. Binder, Thorsten Wiech, Peter F. Zipfel, Christine Skerka

**Affiliations:** 1 Department of Infection Biology, Leibniz Institute for Natural Product Research and Infection Biology, Jena, Germany; 2 Institute of Cell Biochemistry, Hannover Medical School, Hannover, Germany; 3 Clinic for Heart and Visceral Surgery, University Heart and Vascular Center Hamburg, Medical University Hamburg-Eppendorf, Hamburg, Germany; 4 Department of General and Interventional Cardiology, University Heart and Vascular Center Hamburg, Medical University Hamburg-Eppendorf, Hamburg, Germany; 5 German Center for Cardiovascular Research (DZHK) Partner Site Hamburg/Lübeck/Kiel, Hamburg, Germany; 6 Institute of Pathology, Medical University Hamburg-Eppendorf, Hamburg, Germany; 7 Department of Translational Research in Psychiatry, Max Planck Institute of Psychiatry, Munich, Germany; 8 Department of Laboratory Medicine, Medical University of Vienna, Vienna, Austria; 9 Department of Ophthalmology, Charité –University Medicine Berlin, a Corporate Member of Free University, Humboldt-University and the Berlin Institute of Health, Berlin, Germany; 10 Institute for Nutritional Sciences, Friedrich Schiller University, Jena, Germany; 11 Division of Rheumatology, Department of Medicine, University of Colorado Anschutz Medical Campus, Aurora, CO 80045 United States of America; 12 Faculty of Biosciences, Friedrich Schiller University, Jena, Germany

**Keywords:** Genetics, Immunology, Molecular biology, Cardiology, Diseases, Medical research, Molecular medicine, Pathogenesis

## Abstract

Atherosclerotic cardiovascular disease (ACVD) is a lipid-driven inflammatory disease and one of the leading causes of death worldwide. Lipid deposits in the arterial wall lead to the formation of plaques that involve lipid oxidation, cellular necrosis, and complement activation, resulting in inflammation and thrombosis. The present study found that homozygous deletion of the *CFHR1* gene, which encodes the plasma complement protein factor H-related protein 1 (FHR-1), was protective in two cohorts of patients with ACVD, suggesting that FHR-1 accelerates inflammation and exacerbates the disease. To test this hypothesis, FHR-1 was isolated from human plasma and was found to circulate on extracellular vesicles and to be deposited in atherosclerotic plaques. Surface-bound FHR-1 induced the expression of pro-inflammatory cytokines and tissue factor in both monocytes and neutrophils. Notably, plasma concentrations of FHR-1, but not of factor H, were significantly (p < 0.001) elevated in patients with ACVD, and correlated with the expression of the inflammation markers C-reactive protein, apolipoprotein serum amyloid protein A, and neopterin. FHR-1 expression also significantly correlated with plasma concentrations of low-density lipoprotein (LDL) (p < 0.0001) but not high-density lipoprotein (HDL). Taken together, these findings suggest that FHR-1 is associated with ACVD.

## Introduction

Inflammation is a hallmark of many diseases and represents an innate immune response to tissue injury and infection, as well as metabolic stress. Although inflammation is usually followed by clearance and healing processes, sustained immune responses that accompany permanent inflammation can cause serious inflammatory injury in the host. These findings have been observed in patients with atherosclerotic cardiovascular disease (ACVD), a condition that has a substantial negative effect on public health^1^. Innate immune reactions, including those involving complement, recognize modified self surfaces such as oxidized lipids and mark these surfaces for immune reactions. Complement proteins have been localized in atherosclerotic plaques, where they induce opsonization, recruit phagocytic cells, and generate pro-inflammatory mediators^3–5^. According to strong complement activation in atherosclerotic plaques, inhibition of the terminal complement pathway was found to substantially reduce plaque formation and inflammation^6,7^.

Factor H-related protein 1 (FHR-1) is a member of the human complement factor H protein family (its mouse homologue is named FHRE), which consists of complement factor H, a splice variant of factor H (factor H-like-1 (FHL-1)), and five FHR proteins (FHR-1 through FHR-5)^8,9^. In contrast to factor H, FHR-1 cannot act as a cofactor for the cleavage of C3b by factor I or accelerate the dissociation of C3 convertase^8–10^. However, all FHR proteins bind to complement C3b and particularly FHR-1 and FHR-3 compete with factor H for binding to C3b and C3d^11^, an activity previously described as deregulation of factor H^12^. Recently, we identified a new function of FHR-1 outside the complement system. FHR-1 acts as an immune sentinel of oxidized surfaces on necrotic cells by binding to oxLDL and activating NOD-, LRR-, and pyrin domain-containing protein 3 (NLRP3) in monocytes. Upon binding via its C-terminus to the G protein-coupled receptor epidermal growth factor (EGF)-like module-containing mucin-like hormone receptor-like 2 (EMR2), FHR-1 induces the release of pro-inflammatory cytokines^13^. FHR-1 binding to necrotic cells was previously observed in damaged kidney tissues of patients with anti-neutrophil cytoplasmic antibody (ANCA)-associated vasculitis (AAV), with serum concentrations of FHR-1 correlating with disease progression^13^. Because patients with ACVD often have hyperlipidemia, permanent inflammation, and plaques containing oxLDL and necrotic cells, the present study investigated the functions of FHR-1 in greater detail by evaluating its activity in patients with ACVD. It remains unclear, however, whether FHR-1 binds to acellular necrotic cores in plaques of ACVD patients, thereby contributing to plaque vulnerability.

## Results

### Homozygous chromosomal deletion of *CFHR1* protects from ACVD

Homozygous deletion of a chromosomal fragment containing the *CFHR3* and *CFHR1* genes (∆*CFHR3/1*) has been reported to be protective against diseases such as IgAN^14^ and age-related macular degeneration (AMD)^15–17^, but it is a risk factor for atypical hemolytic-uremic syndrome (aHUS)^18,19^. We hypothesized that the protective effect of ∆*CFHR3/1* could be explained by the previously described pro-inflammatory activity of FHR-1^13^ and assessed whether ∆*CFHR3/1* also protects against ACVD. Assessment of a cohort of patients with advanced ACVD showed that the frequency of ∆*CFHR3/1* was 1.2% (3/244), compared with 5.2% (28/525) in a healthy age-matched (> 50 year) control cohort (Table 1). These healthy controls had been included in a case control study of patients with major depression disorders, with this subgroup of healthy individuals evaluated by multiplex ligation-dependent probe amplification (MLPA) assays to determine their ∆CFHR3/1 status^20^. Similarly, the frequency of ∆*CFHR3/1* was found to be 0.8–1.2% in patients with AMD, compared with 4.9–5.2% in control individuals^11,15–17^.

**Table 1:** Description of study cohorts.

	Healthy individuals (n = 525)	ACVD patients (n = 244) Cohort 1	CVD patients (n = 1217) Cohort 2
Age (years), mean (± SD)	61.2 (± 7.8)	68 (± 19)	69 (± 10.6)
Females, number (%)	351 (66.8)	45 (18.4)	326 (26.8)

*CAD* coronary artery disease, *ACVD* atherosclerotic coronary vascular disease.

Because of the relevance of these findings, we determined the frequency of homozygous *CFHR3/1* deficiency in a second, larger cohort of patients with ACVD (CVD, cohort 2), finding a similarly low frequency of homozygous *CFHR1*-deficient patients (16/1217, 1.3%; Table 2) when compared with an age-matched control group (Table 1). Except for one patient (0.08%), all *CFHR1* deficient patients also showed homozygous deficiency of *CFHR3*. In total, non-deficiency resulted in an approximately 4.5-fold higher risk of developing ACVD. Taken together, these new findings indicate that ∆*CFHR1* is associated with protection against ACVD.

**Table 2:** Homozygous *CFHR1* deficiency in patients and healthy individuals.

	Number of subjects	Homozygous *CFHR1* deletion n (%)	Odds ratio* 0.05	p-value
Healthy individuals	525	28 (5.2)		
ACVD cohort 1	244	3 (1.2)	4.5	0.0054
CVD cohort 2	1217	16 (1.3)	4.3	0.0001

*CVD* coronary vascular disease, *ACVD* atherosclerotic coronary vascular disease.

*Fold increased risk in non-*CFHR1*-deficient individuals. Chi-square test, Fisher’s exact test.

### FHR-1 binds to atherosclerotic plaques and stimulates blood monocytes and neutrophils

**Figure 1:**
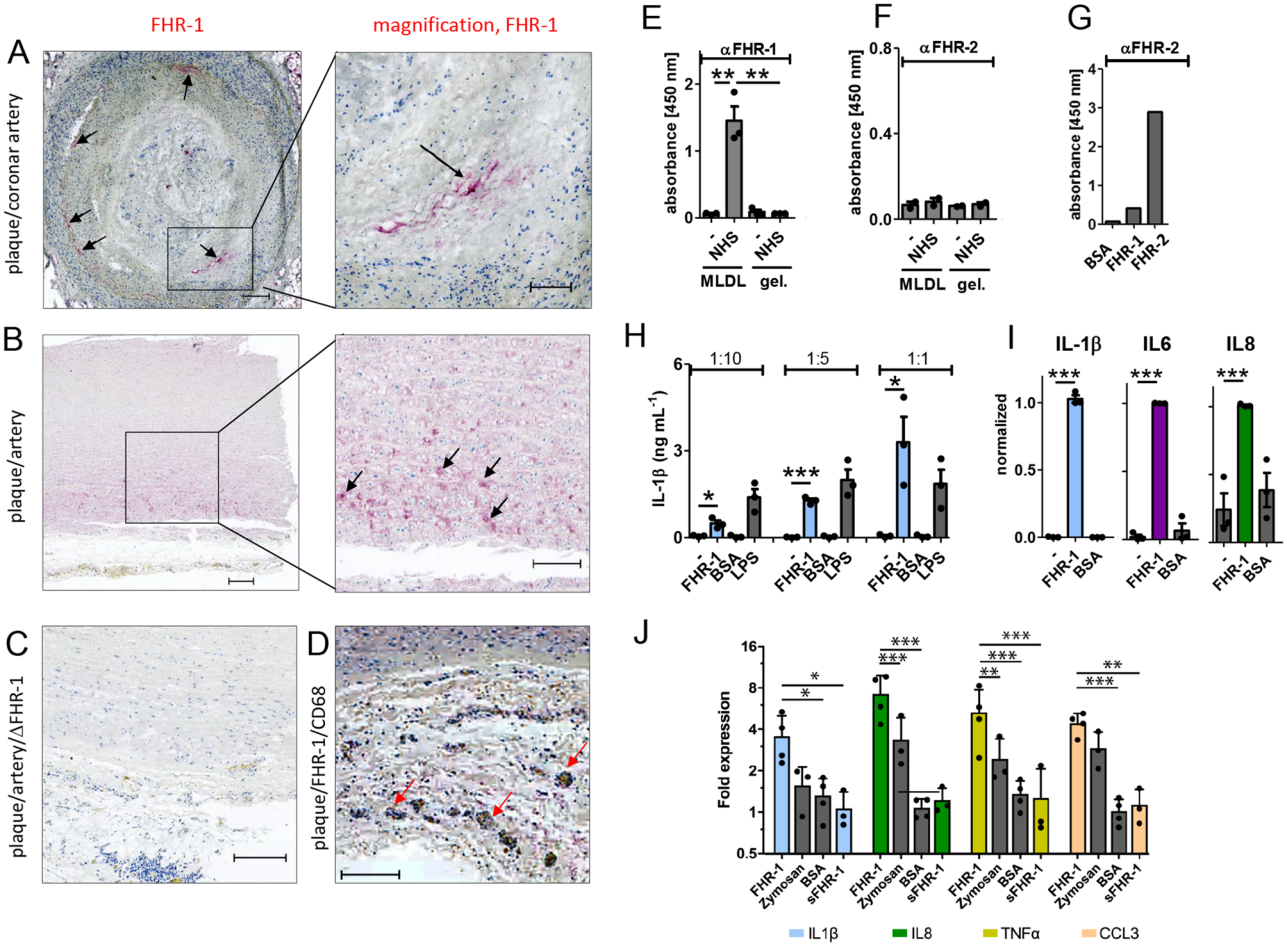
FHR-1 binds to atherosclerotic plaques and is pro-inflammatory. (**A**) Immunohistochemical positivity for FHR-1 (red/arrows) in necrotic/degenerated areas of an atherosclerotic plaque of a coronary artery **(B)** and in the media of aortas with mucoid degeneration **(C)** of ACVD patients but not ∆*CFHR3/1* ACVD patients. (**D**) FHR-1 co-localization with macrophages (arrows). **(A–D)** bars = 200 µm; each picture is representative of three patients with and without FHR-1. Binding of serum **(E)** FHR-1, but not **(F)** FHR2, to MDA-LDL (MLDL). (**G**) Detection of FHR-2 but not FHR1 by monoclonal antibody to FHR-2 mAb. *gel* gelatin. **(H)** Immobilized FHR-1 induces IL-1β secretion in whole human blood (diluted 1:10, 1:5, or 1:1), lipopolysaccharide (LPS) = control activator **(I)** Immobilized FHR-1 induces macrophage secretion of IL-1β IL-6 and IL-8 (BSA = bovine serum albumin) **(J)** upregulates expression of IL-1β, IL-8, TNFα, and CCL3 genes in neutrophils (zymosan = control activator; sFHR-1 = soluble FHR-1). Data in **(E)**—represent the mean ± SEM of three independent experiments with different donor cells. *p ≤ 0.05, **p ≤ 0.01, ***p ≤ 0.001 by unpaired two-tailed t-tests.

FHR-1 was found to localize to necrotic cells (Fig. 1A,B) in coronary artery plaques, but was not detected on healthy tissue adjacent to these plaques or on tissue derived from ∆*CFHR3/1* patients (Fig. 1C). FHR1 co-localized with macrophages, as shown by co-staining with antibody to CD68 (Fig. 1D). Consistent with this observation, recombinant and serum FHR-1^13^, but not FHR2 derived from normal human serum (NHS), bound to oxidation-specific epitopes such as malondialdehyde-modified LDL (MDA-LDL; Fig. 1E,F) as we previously reported^13,20^. The detection of FHR2 was confirmed by ELISA (Fig. 1G). The pro-inflammatory functions of FHR-1 were evaluated using an ex vivo whole blood system, with FHR-1 triggering IL-1β release in whole blood (Fig. 1H). Both isolated primary blood monocytes^13^ and monocyte-derived macrophages secreted pro-inflammatory cytokines and chemokines (IL-1β, IL-6, and IL-8) upon incubation with immobilized FHR-1 (Fig. 1I). In addition, immobilized but not soluble FHR-1 activated blood-derived neutrophils, with RT-qPCR showing that the levels of expression of IL-1β, IL-8, tumor necrosis factor-alpha (TNFα and the chemokine CCL3 were significantly upregulated (Fig. 1J).

Because of the central role of monocytes in ACVD and our observation that FHR-1 promotes inflammation in monocytes and neutrophils, we assessed whether FHR-1 bound to necrotic surfaces, as is found in the necrotic core of atherosclerotic plaques, induces inflammation. Oxidation of LDL particles was reported to be an initial event in atherosclerotic plaque and foam cell formation, and to be involved in various pro-inflammatory mechanisms, including NLRP3 inflammasome activation^21^. FHR-1 binds to oxLDL and seems to act as a guardian of oxidized surfaces to induce immune responses. Components deposited within atherosclerotic lesions, such as cholesterol crystals^22,23^, oxLDL^21,24,25^, and complement, have been reported to induce inflammation^26^.

### FHR-1 circulates in human and murine blood plasma on extracellular vesicles

**Figure 2:**
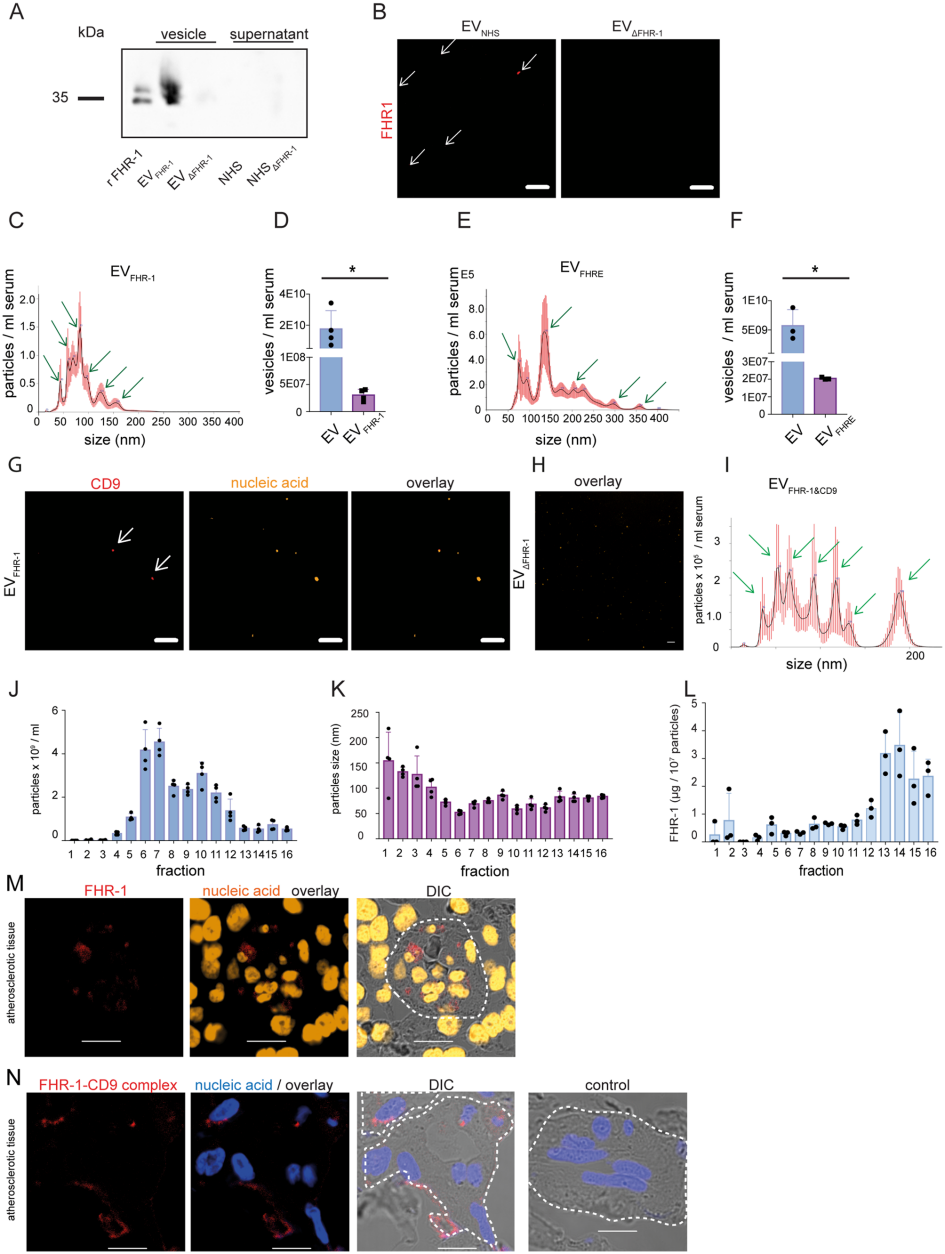
FHR-1 and FHRE circulate on extracellular vesicles (EVs) in normal human serum (NHS) and normal mouse serum (MS). **(A)** Detection of FHR-1 in the EV fraction from NHS (EV_NHS_) but not from homozygous FHR-1-deficient (EV_ΔFHR-1_) human serum by western blot analysis. FHR-1 was also absent from the supernatant fractions of both sera. Results shown are representative of three experiments. An uncropped gel is shown in Supplementary Information. **(B)** Tracking of EV_NHS_ and EV_ΔFHR-1_ by live-cell imaging using CLSM. EVs were stained with anti-FHR-1 antibodies conjugated with Alexa Fluor 647 (red). Bars: 10 µm. **(C–F)** Size distribution and number of vesicles isolated from EV_FHR-1_ and EV_FHRE_ determined by DLSM using NanoSight NTA 3.2 software. Graphs in (**C,E**) represent overlays of results from 3 to 4 donors. (**(C)**, SEM ± standard error, *p ≤ 0.0255 by unpaired two-tailed t-test, n = 4), and (**(E)**, SEM ± standard error, *p ≤ 0.0232 by unpaired two-tailed t-test, n = 3). **(G)** Tracking by live-cell imaging using CLSM of EV_FHR-1_ and EV_ΔFHR-1_ stained with anti-CD9 antibody (vesicle marker) and SYTOX orange (nucleic acid marker), Bars: 10 µm. **(H)** Size distribution of EVs transporting CD9 and FHR-1 (EV_CD9&FHR-1_). EV_FHR-1&CD9_ were captured with anti-CD9-coated beads from EV_FHR-1_, isolated from 1 mL NHS and analyzed by DLSM using NanoSight NTA 3.2 software. **(I)** Particle numbers **(J)** and sizes **(K)** in fractions obtained by size-exclusion chromatography of EV_NHS_ measured by DLSM. **(L)** High FHR-1 content in fractions 13–16 determined by ELISA. FHR-1 (red) **(M)** and FHR-1, and vesicle marker CD9, were in close proximity (red) **(N)** in atherosclerotic tissues, mainly in blood vessels (stippled lines). Complexes were analyzed by proximity ligation assays using anti-FHR-1 and anti-CD9, and were detected by CLSM. EV_FHR-1_ carry nucleic acids (orange). Bars = 10 µm.

Because FHR-1 was previously described as being a constituent part of lipoprotein particles^27^, we evaluated whether FHR-1 is transported on extracellular vesicles (EVs). EVs were therefore isolated from normal human serum as well as from FHR-1-deficient human serum (NHS_ΔFHR-1_) by polymer precipitation. EVs were separated from the supernatant, and both were immunoblotted with an anti-FHR-1 antibody. EVs from NHS but not from NHS_ΔFHR-1_ were positive for FHR-1 signals in the form of two typical glycosylated bands of 37 and 41 kDa, similar to recombinant FHR-1 (Fig. 2A). The presence of FHR-1 on EVs was confirmed by staining EVs with fluorescently labeled anti-FHR-1 (Fig. 2B). EVs ranged in size from 50 to 200 nm, as measured by dynamic light scattering microscopy (DLSM) (Fig. 2C). Thus, FHR-1 is likely to be transported on exosomes, which range between 30 and 150 nm in diameter and originate from multi-vesicular bodies. EVs carrying FHR-1 (EV_FHR-1_) were isolated from this vesicle population by immune separation, with one of the 570 EVs isolated from NHS found to transport FHR-1 (Fig. 2D). Similar results were obtained with EVs isolated from mouse serum. The EVs ranged in size from 50 to 300 nm, whereas most of the FHRE-transporting vesicles ranged in size from 50 to 250 nm (Fig. 2E), with one of the 280 EVs transporting FHRE (Fig. 2F).

To confirm that FHR-1 transporting EVs (EV_FHR-1_) captured with anti-FHR-1 coated beads indeed represent EVs, EV_FHR-1_ were stained with the vesicle marker anti-tetraspanin (CD9) and with SYTOX orange, a nucleic acid stain that permeates EVs and stains EVs transporting small RNAs. Fluorescent live-cell imaging showed CD9 and nucleic acid staining, confirming that FHR-1 is located on nucleic acid-transporting EVs (Fig. 2G), in contrast to EVs isolated from NHS_∆FHR1_. (Fig. 2H). Capture of CD9 vesicles from EVs transporting FHR-1 with anti-CD9 coated beads revealed that these EVs transported both CD9 and FHR-1 (EV_FHR-1&CD9_), with these EVs ranging in size from 30 to 200 nm as measured by DLSM (Fig. 2I). These findings confirm that FHR-1 is transported on extracellular levels.

To further characterize EV_FHR-1_, EVs were isolated from NHS by size-exclusion chromatography, and particles obtained in 16 fractions were investigated by DLSM. Fractions 5–9 contained the largest number of particles (2–4 × 10^9^ particles mL^−1^; Fig. 2J), with these particles ranging in size from 150 (fraction 1) to 50 nm (fraction 6) (Fig. 2K). Measuring the FHR-1 concentration in each fraction by ELISA revealed FHR-1 transporting vesicles in fractions 12–16 (Fig. 2L), with the highest FHR-1 concentrations detected in fractions 12–6 (1.2–3.3 µg/10^7^ particles) containing small particles 50–75 nm in size. These results confirm that FHR-1 is associated with small EVs, likely exosomes. These vesicles were also found in stained tissue sections from atherosclerotic patients.

Proximity ligation assays (PLA) of both FHR-1 alone (Fig. 2M) and FHR-1-CD9 complex (Fig. 2N) showed staining signals in blood vessel structures (stippled lanes) of atherosclerotic tissue sections.

### FHR-1 concentrations are elevated in ACVD patients and correlate with disease markers

**Figure 3:**
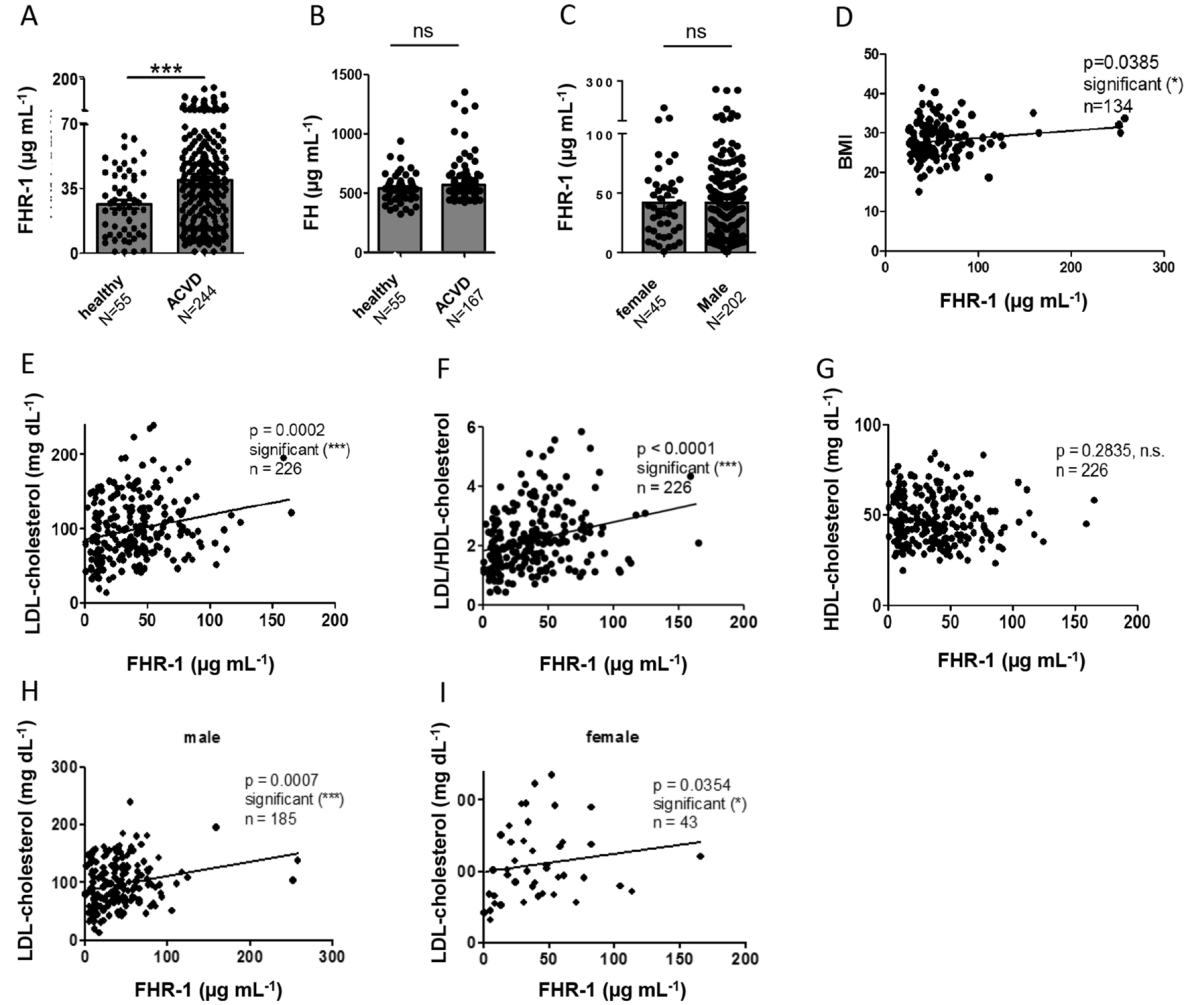
Elevated serum FHR-1 concentrations in ACVD patients correlate with low-density lipoprotein (LDL)-cholesterol concentrations. **(A)** FHR-1 concentrations were significantly higher in ACVD patients than in healthy individuals, **(B)** whereas Factor H concentrations were similar in the two groups. **(C)** Equal elevation of FHR-1 concentrations in men and women with ACVD. **(D–G)** Correlations of FHR-1 concentrations with **(D)** body mass index (BMI), **(E)** LDL concentrations, and **(F)** LDL/HDL ratios, but not **(G)** HDL concentrations, in patients with ACVD. **(H,I)** Correlations of FHR1 and LDL concentrations in **(H)** men and **(I)** women with ACVD. Data in **(A–C)** are reported as mean ± SEM and compared by unpaired two-tailed t-tests with Welch’s correction. Data in **(D–I)** represent Spearman’s correlations, *p ≤ 0.05, ***p ≤ 0.001.

To determine whether FHR-1 contributes to inflammation in ACVD, serum FHR-1 concentrations were measured in 244 patients (cohort 1; Table 1) with advanced ACVD awaiting bypass surgery, and in healthy subjects. FHR-1 concentrations were significantly higher in ACVD patients than in healthy individuals (39.2 ± 1.9 μg mL^−1^ vs. (26.5 ± 2.3 μg mL^−1^, p < 0.0001; Fig. 3A) in contrast to complement factor H (Fig. 3B). FHR-1 concentrations were similar in men and women with ACVD (Fig. 3C), with FHR-1 concentrations above normal (26.5 μg mL^−1^) correlating with patient body mass index (BMI) (Fig. 3D). Higher blood concentrations of LDL-cholesterol and higher LDL- to HDL-cholesterol ratios have been reported to increase the risk for ACVD^2^. The present study found that FHR-1 concentrations significantly correlated with LDL concentrations and LDL/HDL ratios, but not with HDL concentrations (Fig. 3E–G), suggesting a close association between FHR-1 and atherosclerosis. FHR-1 concentrations correlated with LDL concentrations in both men and women with ACVD (Fig. 3H,I).

**Figure 4:**
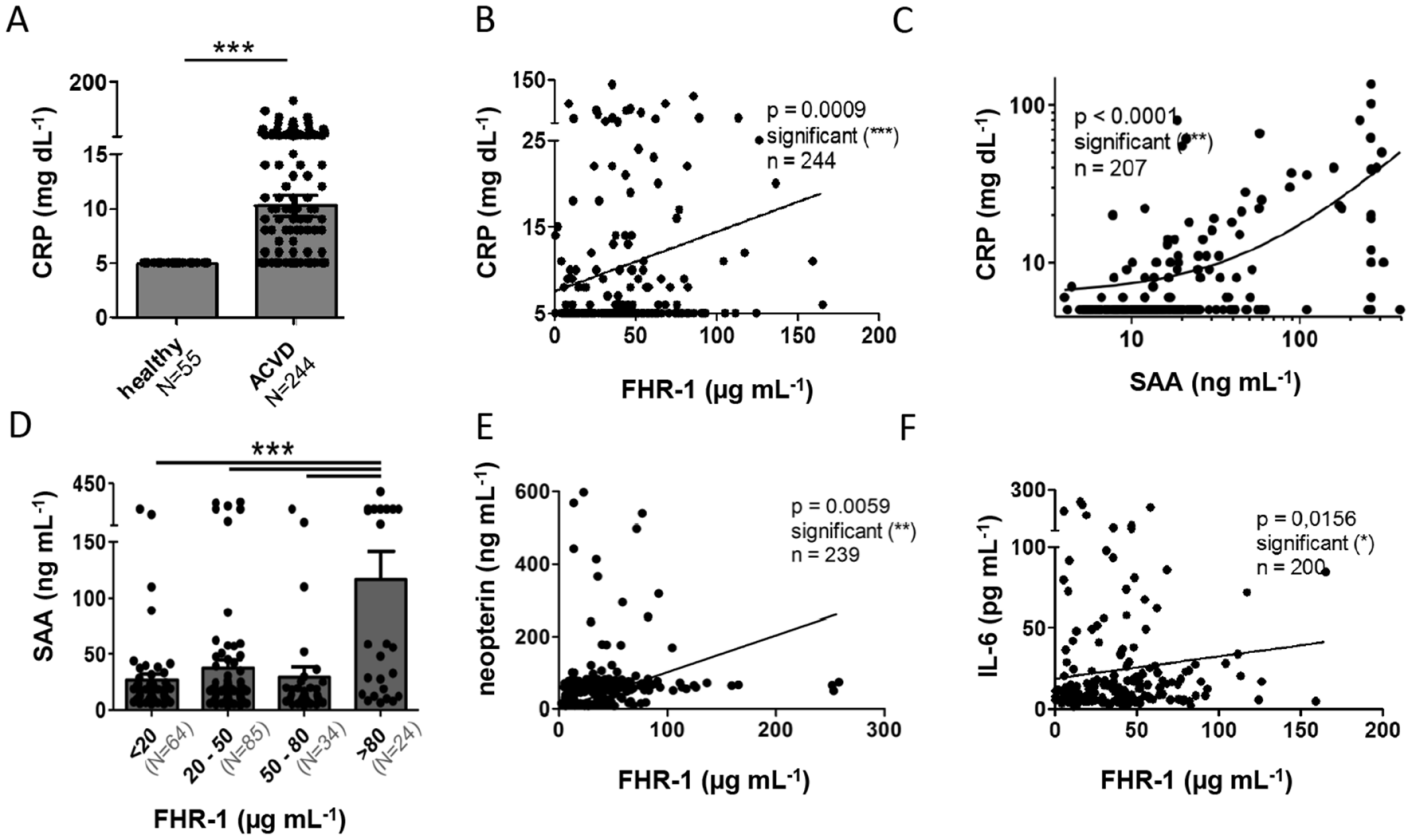
Elevated serum FHR-1 concentrations in ACVD patients correlate with inflammation markers. **(A)** C-reactive protein (CRP) concentrations were significantly higher in ACVD patients than in healthy controls (mean ± SEM, unpaired two-tailed t-test). **(B)** Correlations of FHR-1 concentrations with CRP concentrations and of **(C)** SAA concentrations with CRP concentrations. **(H)** ACVD patients with high FHR-1 levels (> 80 µg mL^−1^) show elevated SAA concentrations (mean ± SEM, unpaired two-tailed t-test), which correlate with **(I)** neopterin and **(J)** IL-6 concentrations. Data in **(B,C,I,J)** represent Spearman’s correlations.

The findings, that FHR-1 induces inflammation in vitro and correlates with inflammation in AAV^13^, suggested that FHR-1 could induce inflammation in patients with ACVD. Concentrations of the acute phase protein C-reactive protein (CRP) were higher in ACVD cohort 1 (10.3 ± 1.0 mg dL^−1^) than in the healthy donors (below the detection limit of 5 mg dL^−1^; Fig. 4A), with FHR-1 concentrations positively correlating with CRP concentrations (p < 0.001; Fig. 4B) and CRP concentrations positively correlating with concentrations of serum amyloid A (SAA), an acute phase protein^28^ (p < 0.001; Fig. 4C). Furthermore, ACVD patients with FHR-1 levels > 80 µg mL^−1^ showed increased levels of SAA (Fig. 4D). FHR-1 concentrations also positively correlated with concentrations of neopterin (Fig. 4E), a marker of cellular immune system activation^29^. Because IL-6 is the major inducer of CRP and SAA, its concentration was also analyzed, with a significant correlation observed between serum FHR-1 and IL-6 levels (p < 0.05; Fig. 4F). These findings suggest that FHR-1 is an active mediator (marker) of inflammation.

### FHR-1 increases tissue factor (TF) expression in monocytes

**Figure 5:**
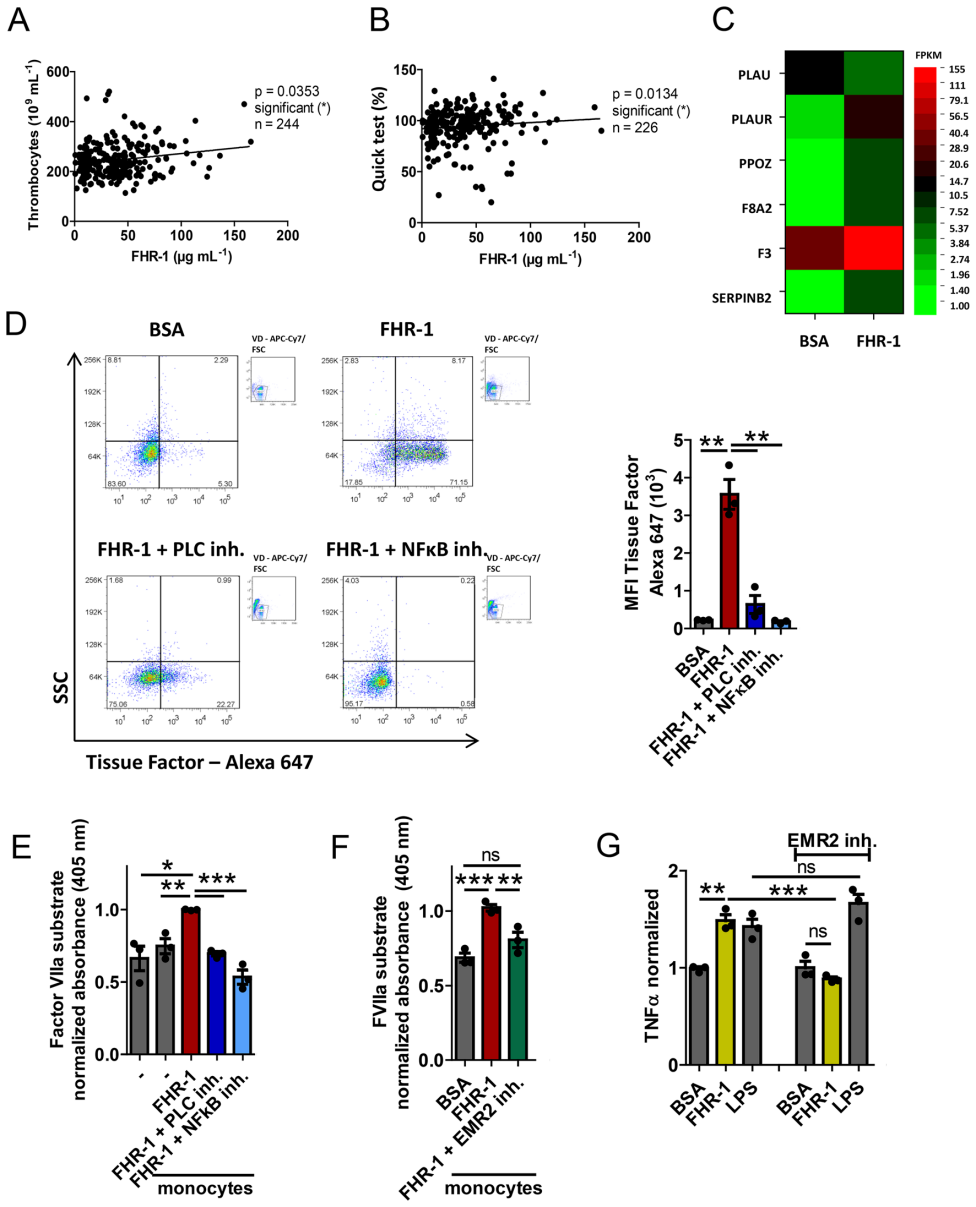
FHR-1 activates the coagulation pathway. **(A)** Correlation between serum FHR-1 concentrations and numbers of thrombocytes and **(B)** Quick test in atherosclerosis patients (Spearman’s correlation). **(C)** FHR-1 induction of the expression of genes encoding proteins in the coagulation pathway in monocytes (n = 4 donors). **(D)** FHR-1-treated monocytes express tissue factor on their surfaces. Representative dot plots from one of three donors are shown. *VD* viability dye. Quantitative analysis of TF expression following inhibition of PLC and NF-κB (right panel). **(E)** FHR-1-treated monocytes show cleavage of Factor VIIa substrate, which is blocked by inhibitors of PLC and NFκB or **(F)** EMR2. **(G)** EMR2 inhibition blocks FHR1-induced, but not LPS-induced, TNFα secretion by monocytes. Data in **(D–G)** represent the mean ± SEM of three independent experiments with cells from different donors. *p ≤ 0.05, **p ≤ 0.01, ***p ≤ 0.001 (unpaired two-tailed t-test).

Inflammatory mediators have been shown to play pivotal roles in thrombus formation, a severe complication of atherosclerosis, as well as in inflammation^2,28–30^. Therefore, the involvement of FHR-1 in coagulation was evaluated. The number of thrombocytes positively correlated with FHR-1 concentration in ACVD patients (p < 0.005, cohort 1; Fig. 5A) as well as Quick test (Fig. 5B) suggesting the involvement of FHR-1 in thrombus formation. To confirm whether FHR-1-treated monocytes directly activate the coagulation cascade, we evaluated the activation of signaling pathways by RNA sequencing in monocytes incubated for 4 h with immobilized FHR-1 or bovine serum albumin (BSA). Several genes involved in the coagulation cascade, including the gene encoding TF, also called factor III and F3, were found to be significantly upregulated in FHR-1-treated monocytes (Fig. 5C).

Flow cytometry showed that in vitro treatment of blood-derived monocytes with FHR-1 resulted in a substantial increase in cell surface TF expression. This effect was inhibited by the addition of pharmacologic inhibitors of phospholipase C (PLC) or NF-κB (Fig. 5D), the major signaling molecules downstream of FHR-1. To determine whether FHR-1-induced TF on monocytes is functionally active, monocytes were treated with FHR-1 for 20 h, washed, and then incubated with a chromogenic factor VIIa substrate, which is cleaved by TF. FHR-1-treated monocytes showed increased cleavage of Factor VIIa compared with untreated monocytes (Fig. 5E). In addition, inhibition of PLC or NF-κB significantly reduced Factor VIIa cleavage by FHR-1-treated monocytes (Fig. 3E).

FHR-1 has been shown to activate the PLC pathway via the G protein-coupled receptor EMR2. EMR2 inhibition for 4 h also reduced the cleavage of Factor VIIa (Fig. 5F) and inhibited TNFα expression (Fig. 5G) in FHR-1-treated monocytes These results show that, in addition to pro-inflammatory genes, FHR-1 induces the expression in monocytes of TF, a protein involved in coagulation.

## Discussion

**Figure 6:**
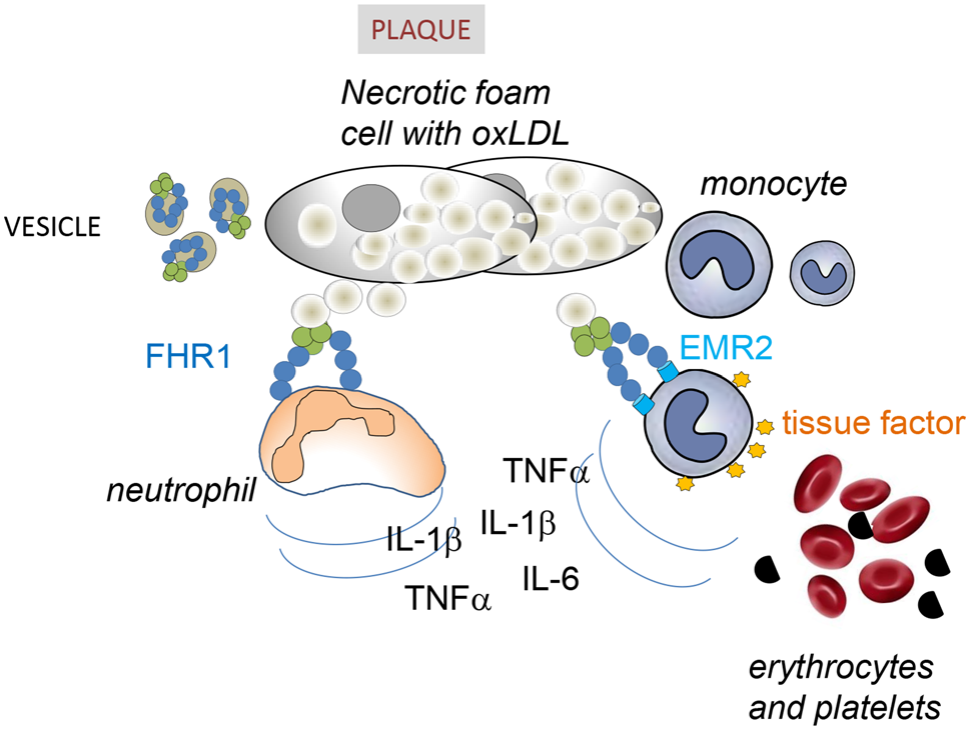
Model of FHR-1-induced inflammation. Extracellular vesicles that transport FHR-1 (EV_FHR1_) bind to oxLDL in necrotic cores of atherosclerotic plaques and activate neutrophils and monocytes. These monocytes and neutrophils release inflammatory cytokines, which attract more immune cells. FHR-1 also induces monocytes to express tissue factor on their surfaces, which can trigger the coagulation pathway and thrombosis, the ultimate complication of atherosclerosis.

Inflammation is a hallmark of many diseases and represents an innate immune response to tissue injury, infection, and metabolic stress. FHR-1 has been shown to act as an immune sentinel by binding specifically to necrotic cell surfaces and inducing pro-inflammatory cytokine release in monocytes. The present study showed that FHR-1 is associated with ACVD. Plasma concentrations of FHR-1 were higher in ACVD patients than in normal individuals and significantly correlated with LDL- but not HDL-cholesterol concentrations in both men and women patients with ACVD. Moreover, homozygous deficiency of the *CFHR1* gene, which encodes FHR-1, was significantly less frequent in two cohorts of ACVD patients than in control individuals. Monocytes bearing FHR-1 were found to induce neutrophils to express pro-inflammatory cytokines. By binding to surfaces such as atherosclerotic plaques, this plasma protein is expected to activate monocytes/macrophages and neutrophilic granulocytes in ACVD patients, inducing inflammation and exacerbating this chronic disease (Fig. 6).

Inflammation is a fundamental multistep and highly regulated process for fighting infections, repairing tissue injury, and re-establishing homeostasis. Although neutrophils and monocytes also cause cell damage and necrosis, their phagocytic actions and interaction are essential for proper regeneration. However, sustained immune responses in patients with persistent inflammation can lead to serious inflammatory injury. In ACVD, pro-inflammatory cytokines enhance the infiltration of immune cells into plaques and predispose vessels to future thromboembolic events^31^. The present study demonstrated that surface-bound, but not circulating, FHR-1 triggers neutrophilic granulocytes to upregulate the transcription and release of pro-inflammatory cytokines such as IL-1β, IL-8, and TNFα. FHR-1 bound to necrotic cells induces inflammation in human blood-derived monocytes by interacting with monocytes via the EMR2 receptor. The present study confirmed FHR-1 stimulation in a whole blood model.

FHR-1 is a dimeric protein circulating in plasma with functions within and independent of the complement cascade. FHR-1 binding to C3b competes with factor H binding to C3b, reducing the inhibitory activities of factor H through a process called deregulation^11,12^. Moreover FHR-1 was shown to inhibit the activation of terminal pathway proteins in vitro^10^. FHR-1, but not FHR-2, on necrotic cell surfaces with exposed oxidized lipids, as in atherosclerotic plaques, activates NLRP3 in monocytes^13^. Evidence showing that FHR-1 is localized to atherosclerotic plaques is consistent with its binding to modified or damaged surfaces.

The present study also showed that FHR-1 is transported through the blood on EVs, likely exosomes. Exosomes, which originate from multi-vesicular bodies, range in size from 30 to 100 nm, but show a considerable overlap with microvesicles 100–1000 nm in size^32^. No FHR-1 signal was detected in supernatants collected after vesicle isolation, indicating that FHR-1 is predominantly associated with vesicles rather than circulating as a soluble protein in plasma. As expected, vesicles isolated from NHS∆_FHR-1_ lacked FHR-1 signals, confirming the specificity of the isolated exosomes. EV_FHR-1_ carry vesicle markers such as CD9 and transport nucleic acids, likely miRNAs. CD9 and miRNA are EV- but not lipoprotein-specific. FHR-1 was reported to be a constituent of HDL blood particles composed of phospholipids, apoA-I, the LPS binding protein LBP, and FHR-2, of average diameter 11.4 ± 12 nm^27^. Although additional studies are required to determine whether FHR-1 is a constituent of both LDL and HDL particles, FHR-1 concentrations did not correlate with HDL concentrations in blood samples. EV contaminants may be present in HDL samples, a finding supported by the presence of CD9 in mass spectrometry studies of HDL samples^33,34^. Because of its ability to bind to artificial vesicles, nonlipidated plasma apoA-I may be associated with EVs^35^. The present study clearly demonstrates that these vesicles contain FHR-1, in addition to factor H^36,37^. Because FHR-1 in NHS is not inflammatory, FHR-1 on exosomes is expected to be in an inactive form but likely becomes activated due to a conformational change upon binding to necrotic surfaces. Indeed, serum-derived FHR-1 has been shown to induce inflammation upon binding to necrotic surfaces^13^. Exosomes are released by many different cells and, as transport carriers, participate actively in intercellular communications^32,35,38^.

Upon binding to damaged surfaces, FHR-1 triggers inflammation and likely upregulates *CFHR1* gene expression in liver cells. FHR-1 concentrations were significantly higher than in normal controls (p < 0.0001). Serum concentrations of FHR-1, however, have been reported to vary from 25 to 95 μg mL^−1^, depending on the ELISA kit. To date, elevated FHR-1 concentrations have been reported in patients with AAV^13^ and IgAN^39,40^, and correlate with disease progression. FHR-1 was also identified as a biomarker of tuberculosis^41^ and acute cellular rejection^42^, diseases characterized by high levels of cell damage and necrosis.

The significantly higher plasma concentrations of FHR-1 in ACVD patients correlate with the inflammatory markers IL-1β, CRP, SAA, and neopterin. Thus, FHR-1 may represent a general cell death-triggered inflammation marker. FHR-1 is found in atherosclerotic plaques and induces isolated monocytes/macrophages and neutrophils to release pro-inflammatory cytokines, including IL-6, which elevates CRP and SAA expression. Stimulation of human monocytes/macrophages with interferon-y induces the production and secretion of neopterin, an indicator of increased cellular immune reactions and oxidative stress^29^. Monocytes and macrophages in particular play important roles in the pathogenesis of atherosclerosis^43–45^. Monocytes infiltrate plaques during their formation and differentiate into macrophages within these lesions^46,47^. In addition, impaired monocyte infiltration into plaques has been shown to attenuate atherogenesis^48,49^. Moreover, monocytosis is an independent risk factor for ACVD, with monocytes causally linked to the progression of atherosclerotic lesions^4,47,50,51^. Targeting inflammation in ACVD patients with anti-inflammatory drugs, such as canakinumab or cholchicine, had a significant impact on the disease as it reduced the incidence of secondary cardiovascular events, including thrombotic heart failure, as shown in the CANTOS and COLCOT trials^52–55^.

The suggested role of FHR-1 in inflammation is corroborated by studies in ACVD patient cohorts, which revealed that homozygous deficiency of *CFHR1* was less frequent in ACVD patients than in control cohorts. Similarly, the frequency of homozygous deficiency of *CFHR1* was lower in AMD patients (0.8–1.2%) than in control subjects (4.9–5.2%)^11,15,17^. These results are in agreement with results from a study showing that AMD was associated with atherosclerosis, and that a polymorphism in complement factor H (rs1061170) associated with AMD was also associated with an increased risk of myocardial infarction^56,57^. Homozygous *CFHR1* deficiency is, in most cases, accompanied by homozygous *CFHR3* deficiency because of the loss of a chromosomal fragment harboring both genes during homologous recombination^19^. However, in vitro assays showed that immobilized FHR3 did not induce inflammation in human monocytes^13^.

Taken together, these results show that FHR-1 on exosomes binds to atherosclerotic plaques, and that this binding increases the production by monocytes and neutrophils of inflammatory cytokines via NLRP3 activation, enhancing the infiltration of immune cells into plaques. As FHR-1 also correlates with CRP and induces IL-6 production and TF expression, FHR-1 may affect the risks of coagulation and thromboembolic events. However, although FHR-1 serum concentrations are substantially higher in ACVD patients than in controls, and although homozygous chromosomal deletion of CFHR1 is less frequent in two cohorts of ACVD patients than in normal individuals, we cannot exclude the possibility that these results are due, at least in part, to other factors, such as the deregulation of factor H by elevated levels of FHR-1 or a genetic constellation of unknown linkage disequilibrium. Further studies are therefore needed to confirm the above results and to analyze the mechanisms of action of FHR-1. Because inflammation exacerbates atherosclerosis, the inhibition of FHR-1 or its associated signaling pathways should be evaluated to determine whether inhibition of FHR1 would be beneficial to ACVD patients.

## Materials and methods

### Measurement of cytokine concentrations

High-binding ELISA plates (Sarstedt) were coated with 50 µL/well each of FHR-1 (5 µg mL^−1^), BSA (5 µg mL^−1^), or DPBS (-) for 1 h at 37 °C. After washing, the plates were incubated with 100 µL/well each of undiluted (1:1) or diluted (1:5 and 1:10) whole blood; 100 µL/well of 1 × 10^5^ macrophages in RPMI 1640 containing l-glutamine (1%), fetal bovine serum (FBS, 10%), NHS, 10%), and gentamicin sulfate; or 100 µL/well of 1 × 10^5^ monocytes in IMDM containing FBS (10%), NHS (10%), and gentamicin sulfate, with or without stimulation with LPS (5 ng mL^−1^). The EMR2 receptor was blocked with an anti-EMR2 antibody (10 μg mL^−1^; R&D Systems, AF4894). TNFα was measured after incubation for 4 h at 37 °C/5% CO_2_, and all other cytokines were measured after 20 h. Cytokines were measured using human cytokine ELISA kits for IL-1β and IL-6 (Thermo Fisher Scientific), IL-8 (Peprotech), and TNFα (BioLegend), according to the manufacturers’ instructions. Absorbance was measured on a Safire 2 microplate reader (Tecan). Similarly, ELISA plates were coated with 50 µL/well each of FHR-1, zymosan, and BSA (5 µg mL^−1^ each) for 1 h at 37 °C and 5% CO_2_, and the plates were subsequently incubated with 100 µL/well of 3.5 × 10^6^ neutrophils in the appropriate media.

### FHR-1 binding to lipids and proteins

Plates were coated with MDA-LDL (5 µg mL^−1^, Cell Biolabs) or gelatin, blocked, and incubated with PBS or NHS (20%), followed by staining with a monoclonal anti-FHR-1 or anti-FHR-2^56^ antibody (each 1:1000), followed by incubation with horseradish peroxidase (HRP)-labeled mouse IgG (1:1000, Dako). TMB chromogen solution (eBioScience) was added, and the reaction was stopped with 2 M H_2_SO_4_ (Roth). The absorbance of each well at 450 nm was measured on a microplate reader.

### RNA purification and analysis

Plates were coated with FHR-1 (50 µL of 5 µg mL^−1^), EV_SE_ (pooled fractions 6–16 from size-exclusion chromatography diluted 1:5 in DPBS), or BSA (20 µg), followed by incubation for 4 h at 37 °C with isolated monocytes^13^ or neutrophils (1 × 10^5^ each)^57^ in DMEM with or without NHS (10%). RNA was extracted from these cells using total RNA purification kits (Norgen Biotek), followed by synthesis of cDNA using high-capacity RNA-to-cDNA kits (Thermo Fisher Scientific and EURx Universal RNA Purification Kit, E3598). Quantitative PCR was performed using a StepOnePlus Real-Time PCR System (Thermo Fisher Scientific) and EURx SG qPCR Master Mix (Cat. no. E0401), with each reaction containing cDNA derived from 0.25 ng total RNA, as measured on a NanoDrop ND-1000 Spectrophotometer (PeqLab Biotechnologie), PerfeCTA SYBR Green FastMix (Quantabio), and primers amplifying sequences specific to β-actin (forward, 5ʹ-GCTAAGTCCTGCCCTCATTT-3ʹ and reverse, 5ʹ-GCTTGCAGTTAGCCAGGTTC-3ʹ); GNB2L1 (forward, 5ʹ-GAGTGTGGCCTTCTCCTCTG-3ʹ and reverse, 5ʹ-GTACAGGTCTTTGCGGATGT-3ʹ); IL-1β (forward, 5ʹ-CTCTCACCTCTCCTACTCACTT-3ʹ and reverse, 5ʹ-TCAGAATGTGGGAGCGAATG-3ʹ); TNFα (forward, 5ʹ-CCAGGGACCTCTCTCTAATCA-3ʹ and reverse, 5ʹ-TCAGCTTGAGGGTTTGCTAC-3ʹ); IL-8 (CXCL8; forward, 5ʹ-AGGAAGAAACCACCGGAAGG-3ʹ and reverse, 5ʹ-GGCAAAACTGCACCTTCACAC-3ʹ); and CCL3 (forward, 5ʹ-GGCTCTCTGCAACCAGTTCTC-3ʹ and reverse, 5ʹ-CTTCGCTTGGTTAGGAAGATGA-3ʹ), with β-actin and GNBL1 used as loading controls. Data were analyzed with Expression Suite software version 1.1 and StepOne software version 2.3. Sequences were PCR amplified according to the manufacturer’s protocol and included an annealing temperature of 60 °C and 40 cycles on an Applied Biosystems StepOne Plus thermocycler. In addition, RNA from FHR-1-treated monocytes from four different donors was pooled (total, 3.4 μg), and PCR-amplified fragments were sequenced by LC Sciences. Data were analyzed using DAVID version 6.7 and Panther version 9.0 software^13^.

### Flow cytometry

High-binding ELISA plates (Sarstedt) were coated with 50 µL/well each of FHR-1 or BSA (5 µg mL^−1^ each), washed, and incubated with 3 × 10^5^ monocytes/well for 20 h at 37 °C and 5% CO_2_. NF-κB was inhibited with BAY11-7085 (30 µM, Sigma-Aldrich), and PLC with U73122 (10 µM, Abcam). Cells were harvested with trypsin/EDTA and incubated with sheep anti-human TF (1:100, Hematologic Technologies), followed by incubation with Alexa Fluor 647-conjugated donkey anti-sheep polyclonal IgG (1:2000, ab150179, Abcam) and the eFluor 780 viability dye (1:10,000, eBioscience), each for 20 min at 4 °C in DPBS containing 1% BSA. Between steps, the cells were washed twice with DPBS containing 1% BSA, and fluorescence was measured via flow cytometry. Cells were gated according to forward scattering properties and viability dye uptake.

### Factor VIIa substrate test

Plates were coated with DPBS or FHR-1 (5 μg mL^−1^), and 1 × 10^5^ monocytes were added in CM containing 10% NHS with or without addition of the PLC inhibitor U73122 (10 μM, Abcam) or the NF-κB inhibitor BAY11-785 (30 μM, Sigma-Aldrich). After incubation for 20 h at 37 °C in an atmosphere containing 5% CO_2_, the cells were washed twice with DPBS, followed by the addition of Factor VIIa substrate (0,3 mg mL^−1^, Sigma-Aldrich) in DPS containing 20% NHS and Mg^2+^/Ca^2+^ (Biowest). After 5 min, absorbance was measured at 405 nm. For EMR2 blocking, an anti-EMR2 antibody (10 μg mL^−1^, AF4894, R&D Systems) was added, and the cells were incubated for 5 h at 37 °C. The cells were washed twice, followed by the addition of Factor VIIa substrate (0.2 mg mL^−1^, Sigma-Aldrich) in DPBS containing 10% NHS and Mg^2+^/Ca^2+^ (Biowest). After 85 min, absorbance was measured at 405 nm.

### Patients

#### Cohort 1

Blood and tissue samples were collected in 2017–2019 from 244 patients diagnosed with advanced ACVD and awaiting coronary artery bypass surgery at the Clinic for Heart and Visceral Surgery, University Heart and Vascular Center Hamburg, Medical University Hamburg-Eppendorf, Hamburg, Germany (cohort 1). This cohort included 199 men and 45 women, of mean age 68 ± 19 years. These blood samples, as well as blood samples from 55 healthy volunteers, were allowed to coagulate, followed by centrifugation at 2000×*g* for 10 min at 4 °C. Serum samples were decanted and stored frozen in aliquots at − 80 °C. FHR-1 concentrations were determined as previously described^13^.

#### Cohort 2

Subjects were selected from the INTERCATH cohort at the Department of General and Interventional Cardiology, University Heart & Vascular Center, Medical University Hamburg-Eppendorf. The INTERCATH cohort is an ongoing, observational cohort study that has included patients undergoing coronary angiography beginning in January 2015, and has been described in detail previously^58^. In brief, all patients have been characterized according to their medical history including comorbidities, medications, lifestyle, socioeconomic information, and laboratory parameters. Coronary angiograms have been analyzed by experienced cardiologists, with the complexity in patients with confirmed coronary artery disease quantified using the SYNTAX score, which was calculated using the internet-based SYNTAX calculator version 2.10 (www.syntaxscore.com). The present analyses included all patients with available SYNTAX scores but excluded all patients with acute coronary syndrome. Genomic DNA was extracted from buffy coats^59^. After quality control and measurement of DNA concentrations, genomic DNA was stored at − 80 °C until further analyses.

#### Cohort 3 (healthy individuals)

Healthy control individuals who had been analyzed for homozygous gene deficiency were included in a previous case control study (n = 525)^20^. The 525 included subjects were enrolled in this study at the Department of Translational Research in Psychiatry, Max Planck Institute of Psychiatry, Munich, Germany.

### Ethics

After informed consent was obtained, patient data, blood, and tissue samples were collected according to the guidelines of the local ethics committees of the Medical University Hamburg-Eppendorf (approval numbers: PV3162, PV4068, and PV5657); Friedrich-Schiller University, Jena, Germany (approval number: 5071-02/17); and Ludwig Maximilians University, Munich, Germany; and according to the Guidelines of the World Medical Association Declaration of Helsinki.

### Immunohistochemistry

Atherosclerotic plaques were obtained from patients with high-grade aortic sclerosis who underwent concomitant ascending aorta replacement during coronary artery bypass grafting. Human coronary plaques were segmented into 3–4 mm blocks, which were fixed overnight in formalin, decalcified in 0.5 M EDTA (pH 7.2), and embedded in paraffin, as previously described^13^. Tissue section (1 μm) were pre-incubated with a protease solution for 30 min at 40 °C, and incubated with a monoclonal anti-FHR-1 antibody and an anti-CD68 antibody^10^ (1:40.000 each) overnight at 4 °C. After washing, the sections were incubated with an AffiniPure rabbit anti-mouse IgG (H + L; Jackson ImmunoResearch) secondary antibody and an anti-alkaline phosphatase antibody (APAAP complex; MyBioSource), followed by development using ZytochemPlus/POLAP100 (Zytomed Systems). Slides were incubated with fuchsin naphthol As-Bi phosphate substrate solution for 30 min, and nuclei were counterstained with Mayer’s hematoxylin solution (MilliporeX) for 1 min. Staining was quantified using ImageJ or ZEN software (version 2, blue edition).

### Analysis of serum samples

FHR-1 and factor H concentrations in serum samples were determined using human FHR1 (RayBiotech) and factor H (Biomatik) ELISA kits according to the manufacturers’ protocols. The absence of FHR-1 from serum samples was confirmed by western blot analysis and by PCR, as described^19^. CRP concentrations were measured using a Cobas8000 analyzer (Roche Diagnostics) on module C701. The CRP concentrations in all samples below the CRP limit of detection of 5 mg dL^−1^ were defined as 5 mg dL^−1^. SAA concentrations were measured by ELISA (Life Diagnostics, no. SAA-20) according to the manufacturer’s protocol. IL-6 concentrations were determined using the LEGENDplex human inflammation panel 1 (BioLegend) according to the manufacturer’s protocol. Patients who had IL-6 concentrations within the detection range of the kit were used for correlation analysis. BMI; LDL- and HDL-cholesterol concentrations; thrombocyte counts; and Quick test values were determined by routine procedures in the clinical laboratory of the University Hospital Hamburg-Eppendorf.

### Vesicle biochemistry

#### Vesicle isolation

Vesicles were isolated from sterile serum and diluted with RPMI-1640. Vesicles were precipitated using ExoQuick-TC (Systems Bioscience)^60^, according to the manufacturer’s protocol. Both the vesicle precipitates and supernatants were collected and stored at − 20 °C for no longer than 1 month.

EVs were also isolated from 1 mL fresh human serum by size-exclusion chromatography using Sepharose CL-2B (Sigma-Aldrich), as described^61^. Sixteen consecutive 1 mL fractions were eluted from each column and stored at − 20 °C until further processing. Upon reuse, the fractions were thawed on ice.

#### Targeted vesicle isolation

Specific FHR-1-transporting EVs were isolated from the total vesicle population using M-pluriBeads (pluriSelect), according to the manufacturer’s instructions. The beads were coated with monoclonal antibody to human FHR-1^10^. FHRE-transporting vesicles were isolated using M-pluriBeads (pluriSelect) coated with a polyclonal antiserum to mouse FHRE generated by immunization with recombinant FHRE protein^13^. Vesicles carrying both FHR-1 and CD9 were similarly isolated from FHR-1-transporting vesicles with M-pluriBeads (pluriSelect, order no. 19-00900-20) coated with antibody to human CD9. The targeted isolated vesicles were directly processed for further experiments.

#### Vesicle counting

The number and size of the isolated EVs were counted and determined by nanoparticle tracking analysis (NTA) using DLSM (NS300 Malvern). Five videos were captured for each sample at a rate of 24 frames per second (fps), each lasting 60 s, and analyzed with NanoSight NTA 3.2 software. The fractions obtained following size-exclusion chromatography were analyzed in the same manner.

#### Antibody conjugation

For imaging analysis, monoclonal FHR-1 antibodies^10^ were coupled to Alexa Fluor 647 dye (Thermo Fisher Scientific, cat no. A20502) by mixing the dye with the antibody at a 2:1 ratio and incubating the mixture for 30 min at room temperature. Residual dye was removed using desalting columns (CentriPure MINI Spin Columns Desalt Z-25; emp Biotech).

#### Vesicle imaging

Vesicles isolated by precipitation from human serum were incubated with monoclonal anti-human FHR-1^10^ antibody conjugated with Alexa Fluor 647 (1:200) to detect FHR-1. FHR-1-transporting EVs were captured using beads coated with monoclonal FHR-1 antibody and were stained with Alexa Fluor 647-labeled anti-human CD9 antibody (1:100; Novus Biological, cat no. NB500-327). Samples were transferred into 35 mm culture dishes with a glass-bottom (Greiner). To detect nucleic acids in vesicles, the vesicles were incubated with 5 µM SYTOX orange (Thermo Fisher Scientific). Pictures were taken with LSM and analyzed using ZEN 2011 software.

PLA assays were performed to evaluate the co-localization of the vesicle markers CD9 and FHR-1. Vesicles isolated by precipitation from human serum were seeded onto 6.7 mm diagnostic slides coated with poly-l-lysine. The vesicles were fixed with 4% formaldehyde, blocked with Duolink blocking solution (Sigma-Aldrich), and incubated with mouse antibody to human FHR-1^13^ (1:200) and rabbit antibody to human CD9 (1:200; Abcam, cat no. ab92726). PLA assays were performed using the Duolink In Situ Red Starter Kit Mouse/Rabbit (Sigma-Aldrich, cat no. DUO92101) according to the manufacturer’s instructions. Images were captured with LSM 710 fitted with ZEN 2011 software.

#### Western blot analysis

To detect vesicle proteins, samples were resolved in non-reducing buffer (Rotiload 1:4), loaded onto 10% polyacrylamide gels, subjected to electrophoresis, and blotted onto polyvinylidene fluoride (PVDF) membranes (Sigma-Aldrich). FHR-1 was detected by incubating the membranes with a monoclonal antibody to human FHR-1^10^ (1 µg mL^−1^) and a goat anti-mouse immunoglobulin-conjugated HRP secondary antibody (1:1000; Dako, cat no. P044701-2). Pictures were captured with Fusion.

### ELISA

FHR-1 concentrations were measured in size-exclusion chromatography fractions using Human FHR-1 ELISA kits (RayBiotech, cat no. ELH-CFHR1) according to the manufacturer’s instructions. Absorption at 450 nm was measured on a TECAN Safire2 microplate reader.

### Immunohistochemistry and PLA

Paraffin-embedded atherosclerotic plaques were prepared as described. Paraffin-embedded liver sections were deparaffinized by treatment with Roticlear (Carl Roth), 100% ethanol, and 90% ethanol. These tissue samples were boiled in 10 mM Na citrate buffer (pH 6.5) for antigen retrieval and permeabilized with 0.1% saponin (Sigma-Aldrich). Tissues were blocked with normal serum block (Biolegend) and human FcR Blocking Reagent (Miltenyi) diluted in antibody diluent (Carl Roth). Tissue samples were incubated with mouse monoclonal FHR-1 antibody^13^ (1:200) and goat anti-mouse IgG (H + L) conjugated to Alexa Fluor 647 (Thermo Fisher Scientific, cat no. A-21235). Nuclei were stained with SYTOX orange. Images were captured with LSM 710 fitted with ZEN 2011 software.

For PLA, deparaffinized and blocked tissue samples were incubated with mouse anti-FHR-1 antibody^13^ (1:200), with or without rabbit anti-CD9 antibody (1:200; Abcam, cat no. ab92726). PLA assays were performed using the Duolink In Situ Red Starter Kit Mouse/Rabbit (Sigma-Aldrich, cat no. DUO92101) according to the manufacturer’s protocol. Images were captured with LSM 710 fitted with ZEN 2011 software.

### Statistical analysis

Significant differences between groups were analyzed by unpaired two-tailed Student’s t-tests using GraphPad Prism version 5. Atherosclerosis patients were analyzed using unpaired two-tailed t-tests with Welch’s correction. Correlations were analyzed using Spearman’s correlation tests. CAIA studies were analyzed using ANOVA and t-tests. p-values ≤ 0.05 were considered statistically significant. Odds ratios were calculated using GraphPad Prism version 5.

### Study approval

Informed consent was obtained from all subjects and/or their legal guardian(s) and patient data, blood, and tissue samples were collected according to the protocls. The experimental protocols were approved by the local ethics committees of the Medical University Hamburg-Eppendorf (approval numbers: PV3162, PV4068, and PV5657), Friedrich-Schiller University (approval number: 5071-02/17), and Ludwig Maximilians University, and according to the Guidelines of the World Medical Association Declaration of Helsinki.

## Supplementary Information

Supplementary Figure S1.

## Data Availability

The data generated for this study have been deposited at the Gene Expression Omnibus (GEO) under accession code GSE119025. Correspondence and material requests should be addressed to christine.skerka@leibniz-hki.de.
