# Supplementary material for: Factor H-related protein 1 (FHR-1) is associated with atherosclerotic cardiovascular disease
Source: Sci Rep. 2021 Nov 18;11:22511. doi: 10.1038/s41598-021-02011-w (PMC8602345; doi:10.1038/s41598-021-02011-w)
Supplement: Supplementary file 1 — Supplementary Figure S1. [file 41598_2021_2011_MOESM1_ESM.pdf]

Supplementary Information

FACTOR H-RELATED PROTEIN 1 (FHR-1) IS ASSOCIATED WITH ATHEROSCLEROTIC CARDIOVASCULAR DISEASE

Sarah Irmscher[1,2,a], Svante L. H. Zipfel[3,a], Luke D. Halder[1], Lia Ivanov[1], Andrés Gonzalez-Delgado[1], Christoph Waldeyer[4,5], Moritz Seiffert[4,5], Fabian J. Brunner[4,5], Monika von der Heide[1], Ina Löschmann[1], Sonia Wulf[6], Darina Czamara[7], Nikolina Papac-Milicevic[8], Olaf Strauss[9], Stefan Lorkowski[10], Hermann Reichenspurner[3], Michael V. Holers[11], Nirmal K. Banda[11], Tania Zeller[4,5], Elisabeth B. Binder[7], Christoph J. Binder[8], Thorsten Wiech[6], Peter F. Zipfel[1,12], and Christine Skerka[1,*¶]

Supplementary Figure 1

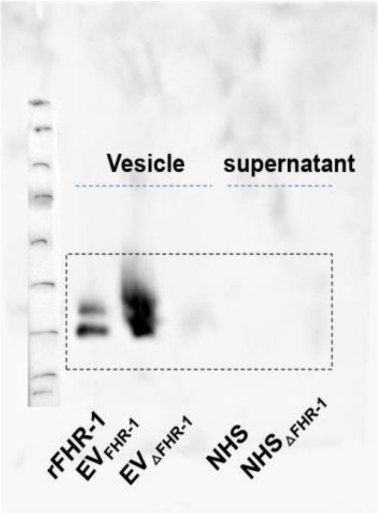

Uncropped WB Figure 2a
